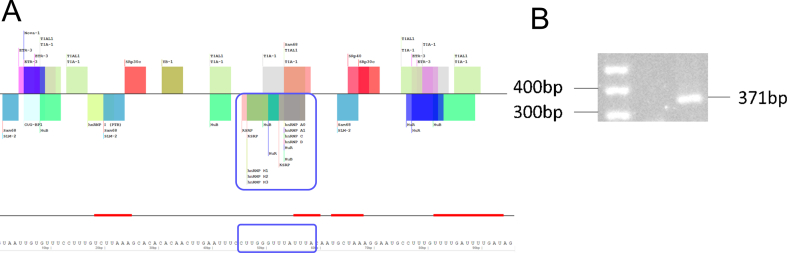# The abnormal splicing regulation network caused by synonymous mutations in *FBN1* exon 39 leads to Marfan syndrome

**DOI:** 10.1016/j.gendis.2024.101371

**Published:** 2024-07-03

**Authors:** Fudan Wu, Mingjie Li, Xuan Zhou, Qianyun Wang, Yan'an Wu

**Affiliations:** a Department of Clinical Laboratory, Xiang'an Hospital of Xiamen University, School of Medicine, Xiamen University, Xiamen, Fujian 361102, China; b Department of Clinical Laboratory, Fujian Medical University Union Hospital, Fuzhou, Fujian 350001, China; c Department of Clinical Laboratory, Mengchao Hepatobiliary Hospital of Fujian Medical University, Fuzhou, Fujian 350000, China

Mutations in fibrillin-1 gene (FBN1) have been shown to be associated with Marfan syndrome and most *FBN1* mutations display missense or nonsense. Recently, reports on synonymous mutation-related diseases have attracted widespread attention. Zhang et al demonstrated that synonymous mutations in saccharomyces cerevisiae have pathogenicity like non-synonymous mutations.^1^ Based on a special Marfan syndrome patient with a novel synonymous mutation in *FNB1*, this study aimed to investigate the pathogenic mechanism of synonym mutation, hoping to explore individualized precision therapy to improve or even cure those diseases caused by synonymous mutations. Through the current study, we naturally come to the following conclusions: mutations within the exon that was away from canonical splicing sites might lead to aberrant splicing, by generating splicing regulatory elements (SRE) and leading to alterations in SRE-binding proteins and thus breaking the splicing balance. Studies on the regulatory process of gene splicing in genetic diseases are expected to provide new prospects for individualized targeted therapy.

We have previously demonstrated that the synonymous mutation site c.4773 was very conservative. Exon skipping still occurred with varying degrees when c.4773A was replaced by the other three nucleotide mutations (G, C, and T).^2^ Based on the synonymous mutation minigene plasmids, synonymous mutations of adjacent bases were built to explore the potential splicing mechanism. Results showed that the MUT-C recombinant plasmid (artificially generated by introducing G > C synonymous mutation at +3 position based on MUT plasmid; Fig. 1A) did correct the wrong splicing when the consecutive multiple Gs of SRE motif was broken.

**Figure 1:**
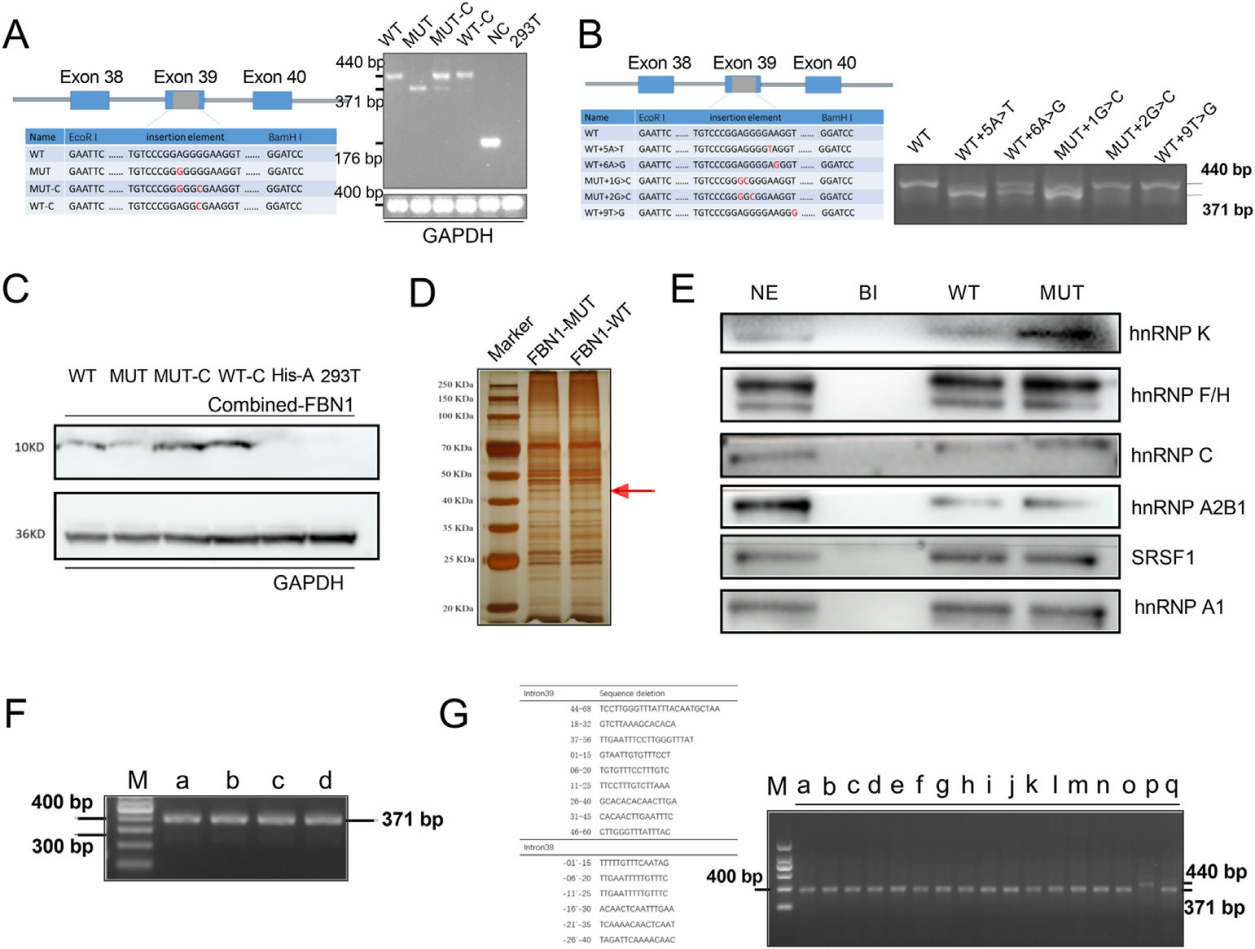
Aberrant RNA splicing regulation of a synonymous mutation c.4773A > G in *FBN1* exon 39. **(A)** The inserted genomic fragments of recombinant plasmids and the bases marked in red are substitutional bases. The electrophoresis results of reverse-transcription PCR. MUT-C group corrects most splicing events (440/371 bp) while WT-C suggests normal splicing (440 bp). **(B)** Electrophoresis diagram of 293T cells transfected with recombinant plasmids. The inserted genomic fragments of recombinant plasmids and the bases marked in red are substitutional bases. WT+5A > T, WT+6A > G and MUT+1G > C groups showed different concentrations of skipped bands (371 bp), accompanied by normal bands (440 bp). WT, MUT+2G > C, and WT+9T > G groups present only a normal band (440 bp). MUT+1G > C group presented two bands, suggesting the mis-splicing has been partially corrected. MUT+2G > C group presented the only normal band, suggesting the mis-splicing is mostly corrected. **(C)** WT and MUT recombined proteins were tested using a Western blot with an anti-6 histidine antibody. Compared with the WT and WT-C groups, the MUT group presented a weaker protein band. MUT-C group, reversing the mis-splicing, presented a normal band. **(D)** The silver-stained proteins pulled down by special RNA probes. **(E)** The splicing proteins were evaluated using RNA pull-down and Western blot assay. The inhibitory splicing proteins (hnRNP K, hnRNP C, and hnRNP A2B1) bound to MUT were significantly increased compared with MUT, while hnRNP F/H suggested a slight increase. hnRNP A1 and SRSF1, which promote splicing, did not differ significantly before and after mutation. **(F)** The mis-splicing event remained uncorrected when hnRNP K and hnRNP C were knocked down. Lanes a and b were si-hnRNP K and si-hnRNP C groups respectively. Lane c was negative control and lane d was the blank 293T cell. All groups presented 371 bp (aberrant splicing events). **(G)** Electrophoresis results of transfected recombinant plasmids with 15 site-directed deletions of partial introns. Lanes a–o on the electropherogram corresponded to the sequence on the left.

Bioinformatics analysis was widely used in the study of RNA splicing. HOT-SKIP tool-based prediction results hinted (Fig. S1) that three mutation hotspots existed at *FBN1* exon 39 that might affect RNA splicing (three artificial plasmids were accordingly built, namely MT, WT+5A > T, and WT+6A > G). After transfected with WT+6A > G plasmid, only about 50% of the band was skipped, while the WT+5A > T was mostly skipped (Fig. 1B), which indicated that clinical symptoms of patients with WT+5A > T mutation might be more severe than those with WT+6A > G. Considering that WT+5A > T is a missense mutation and WT+6A > G is a synonymous mutation, the patient's symptoms might be more damaging to some extent. In brief, while current bioinformatics analyses were convenient and accurate to a certain degree, they could not predict more complex and intrinsic mutational differences. As aforementioned, our results might have a positive meaning for judging the severity and prognosis of patients with these mutations.

It has been reported that the base arrangement of consecutive multiple Gs motifs (three or more) was part of the SRE binding motifs of multiple repressive splicing proteins, often resulting in aberrant exon skipping.^3^ To verify the role of consecutive Gs motif in *FBN1* exon 39, three recombinant plasmids were built (harboring MUT+1G > C, MUT+2G > C, and WT+9T > G). Results showed that the MUT+1G > C plasmid restored part of the splicing, and the MUT+2G > C plasmid almost completely restored normal splicing, suggesting that breaking the consecutive Gs might partially restore the splicing balance. However, it still presented a normal splicing even if the WT+9T > G plasmid produced three consecutive Gs, indicating that there might existed other SRE motifs that were resistant to the abnormal splicing process (Fig. 1B).

The ultimate causative mechanism of genetic disorders is generally considered to be haploinsufficiency or dominant negative effects. In the current study, Western blot results demonstrated that the patient had a lower recombinant protein expression level than the normal control when using an anti-6 histidine antibody. Besides, the MUT-C group, reversing the mis-splicing, presented a normal band (Fig. 1C). In this study, while the exon skipping event produced a truncated mRNA, it did not cause a frameshift and downstream premature termination codon. It might be that the truncated mRNA effortlessly degraded, or the translated truncated protein broke down fleetly, reducing the total amount of physiological fibrillin-1 protein, and thus leading to Marfan syndrome. Therefore, we believed that it was the synonymous mutation (c.4773A > G) causing haploinsufficiency that ultimately led to Marfan syndrome.

Although the synonymous mutation did not produce abnormal fibrillin-1 protein, some changes occurred at the RNA level. Bioinformatics predicted that more elements of splicing silencer would be produced when the base “A" was substituted for “G" at the c.4773 position, and the inhibitory splicing proteins binding to them shall increase accordingly (Fig. S2). The silver staining result determined that there existed significant differences in protein bands pulled down by WT and MUT probes (Fig. 1D). Subsequently, Western blot results suggested that hnRNP K was significantly up-regulated, and both hnRNP C and hnRNP A2B1 were only slightly elevated, while hnRNP F/H were mildly elevated. Nevertheless, serine and arginine-rich splicing factor 1 (SRSF1), promoting normal splicing events, barely changed at all after the mutation occurred. Meanwhile, hnRNP A1, the most common inhibitory splice protein, exhibited no change (Fig. 1E). Taken together, these results suggested that changes in splicing regulatory proteins disrupted the balance of original splicing regulatory networks, thereby inhibiting normal splicing and causing exon skipping.

To do preliminary exploration for gene therapy, we attempted to restore normal splicing regulation by knocking down the inhibitory splicing proteins. A special siRNA targeted hnRNP K and hnRNP C separately was co-transfected with the WT and MUT plasmids, and quantitative reverse-transcription PCR and Western blot results exhibited that hnRNP K and hnRNP C were significantly downregulated (Fig. S3). Still, the mis-splicing event remained uncorrected (Fig. 1F). From our perspective, splicing regulation is a complex network where stimulative splicing proteins and inhibitory splicing proteins are in a delicate equilibrium. Regulations of any or several proteins in both protein families can have an important impact on splicing events. Among various types of splicing proteins, there may be a key factor, but not necessarily hnRNP C and hnRNP K.

Nucleic acid therapeutics, an emerging field aimed at treating human diseases by regulating gene expression, have attracted particular attention due to the successful development of mRNA vaccines for SARS-CoV-2. Investigation on mis-splicing has raised concerns about another nucleic acid treatment, splice-switching oligonucleotides (SSOs). SSOs inhibit gene expression by sequence-specific targeting of precursor mRNAs and have been reported as drugs for the treatment of genetic diseases. The most successful example was the treatment for spinal muscular atrophy.^4^ In the study, an SSO (brand name Nusinersen), specifically targeted SREs, was used to prevent the skipping of SMN2 (survival of motor neuron 2) exon 7, increasing the total amount of intact SMN2, and greatly relieved spinal muscular atrophy. A similar SSO treatment was applied to treat Duchenne muscular dystrophy and familial dysautonomia caused by mutations in IKBKAP (encoding IκB kinase-associated protein). Most of these studies indicated that splicing mutations disrupted normal SREs, affecting the type and affinity of binding hnRNPs and eventually leading to diseases. Given the small number of patients with genetic diseases, individualized therapy is becoming increasingly important and SSO technology can serve as a completely promising platform for personalized treatment.

Based on the experience of the above predecessors in developing related SSO technology. In this study, we first selected a region in the intron 39 that was shown to bind to more inhibitory splicing proteins by the SpliceAid2 tool (Fig. S4) and synthesized a 2′-O-methyl phosphorothioate-modified oligonucleotide targeting this region for blocking, but it did not have the expected effect for restoring normal RNA splicing. Therefore, we hope to expand the screening area of SSOs by gradually removing specific intron sequences flanking the synonymous mutation (where functional SSOs might directly target), and disappointingly, no intron regions capable of correcting splicing have been found (Fig. 1G). As we suspected, it has been reported that blockade at the distant ISS+100 sequence also restores partial splicing events.^5^ It is speculated that the intron sequences that inhibit splicing events might be distributed in distant intron areas where they were not involved in this study. In the future, we may need to continuously extend the intron sub-region to find efficient SSOs or apply special bioinformatics algorithms to find promising SSOs that target the appropriate sequence to rectify the abnormal splicing events.

## Conflict of interest

The authors have no competing interests to declare.

## Funding

This work was supported by the 10.13039/501100017686Fujian Provincial Health Technology Project (China) (No. 2023QNA016), the Guidance in Medical and 10.13039/100018696Health Program of Xiamen, China (No. 3502Z20209120), and Scientific Research Foundation for Advanced Talents, Xiang'an Hospital of 10.13039/501100008865Xiamen University (China) (No. PM201809170018).

## Figures and Tables

**figs1:**
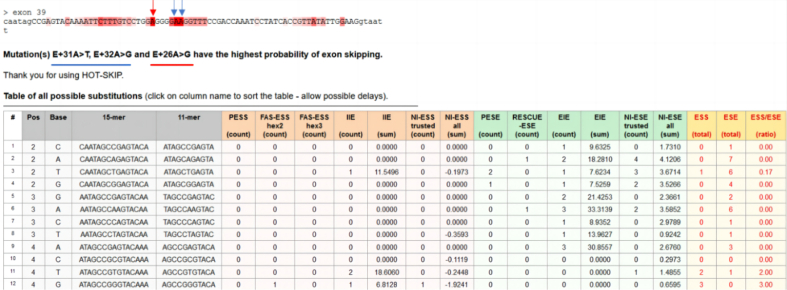


**figs2:**
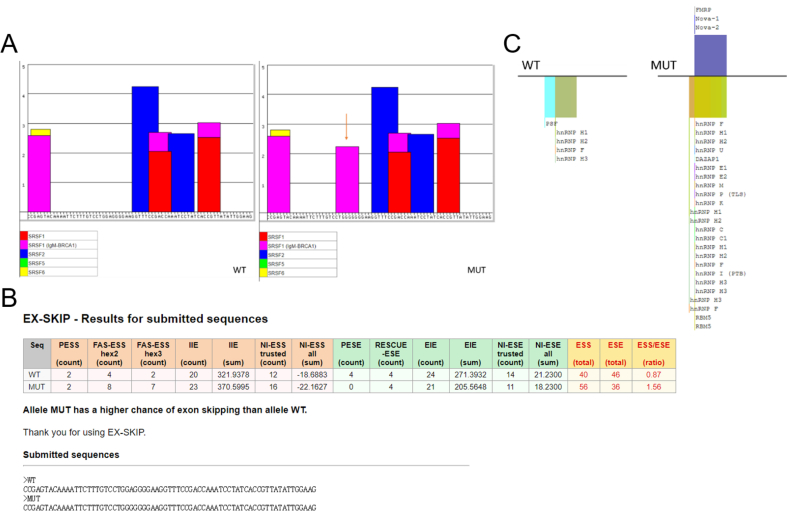


**figs3:**
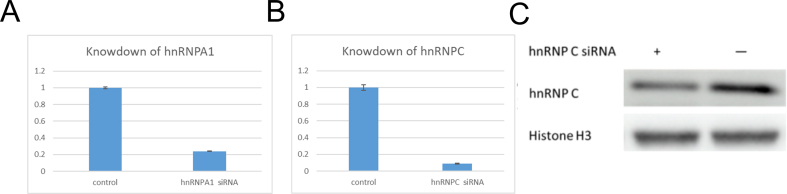


**figs4:**