# Supplementary material for: The abnormal splicing regulation network caused by synonymous mutations in FBN1 exon 39 leads to Marfan syndrome
Source: Genes Dis. 2024 Jul 3;12(3):101371. doi: 10.1016/j.gendis.2024.101371 (PMC11772951; doi:10.1016/j.gendis.2024.101371)
Supplement: Multimedia component 1 [file mmc1.docx]

**Materials and methods**

**Construction and transfection of *FBN1* minigene model**

This study had acquired the approval from ethics committee of Fujian Provincial Hospital (No. K2015-02-021) and adhered to the Declaration of Helsinki. The FBN1 synonymous mutation c.4773A>G was idtified in the proband with MFS.

In order to investigate the effect of the synonymous mutation c.4773 A>G (p. Gly1591Gly) on pre-mRNA splicing, the *FBN1* minigene model was constructed as described. The genomic fragment of 2352 nucleotides from exon 38 to exon 40 (g.182830-185181) was amplified using a nested PCR and then the fragment with restriction sites was cloned into the vector of pcDNA3.1 (-)/myc-His A to build a wild-type (WT) minigene model. Three mutant-type (MUT) minigene models (c.4773A>G, c.4773A>C and c.4773A>T) come from the WT minigene model using site-directed mutagenesis (QuikChange II XL Site-Directed Mutagenesis Kit; Agilent Technologies, Netherlands). Sanger sequencing verifies the accuracy of the inserted sequence. Hela cell was cultured in Dulbecco’ s modified Eagle’s medium (DMEM) supplemented with 10% fetal bovine serum (Gibco, USA) and transiently transfected with 2.5 μg WT or MUT *FBN1* minigene construct using Lipofectamine3000 (Invitrogen, USA). Cells were harvested in TRIzol Reagent (Invitrogen, USA) and then total RNA was extracted and reverse-transcribed by reverse transcription-polymerase chain reaction (RT-PCR). PCR products were subsequently analyzed by agarose gel electrophoresis (AGE) and Sanger sequencing.

**RNA-pull down**

The affinity purification of RNA-binding proteins was performed with 3-biotin-coupled RNA oligonucleotides (Sangon Biotech, China). The sequences of the RNA oligonucleotides, comprising FBN1 position c.4773 were: 5’-UUGUCCUGGAGGGGAAGGUUU-3’-Biotin (FBN1-WT), 5’-UUGUCCUGGGGGGGAAGGUUU-3’-Biotin (FBN1-MUT). For each purification test, 100pmol RNA oligonucleotide was coupled to 100μl streptavidin coupled magnetic beads (Invitrogen, US) and incubated with HeLa nuclear extract (Cilbiotech, Mons, Belgium). After washing, bound proteins were investigated by western blotting using a monoclonal mouse antibody toward SRSF1 (SF2/ASF), hnRNP A1, hnRNP A2/B1, hnRNP K, hnRNP C (Abcam，USA) or polyclonal antibodies toward hnRNP F/H (sc-10029 from Santa Cruz Biotechnology, Santa Cruz, USA).

**Western Blot**

Protein samples separated by 12% SDS-PAGE were electroblotted onto nitrocellulose membranes. The blots were then probed with monoclonal antibodies anti-hnRNP A/B family (A1/UP1-62), anti-hnRNP A1 (A1/UP1-55, Abcam, USA), and anti-hnRNP A2/B1 (DP3B3, Abcam, USA), followed by horseradish peroxidase-conjugated goat anti-mouse secondary antibody (Pierce). Protein signals were detected with Lumi-Light Western Blotting Substrate (Roche Diagnostics, USA).

**Knockdown of hnRNP A1 and hnRNP C**

The special spliced genes were knocked down in order to restore the normal splicing events in HEK293 and HeLa cells. Approximately 150,000 cells were seeded in each well in a six-well plate and 30 pmol hnRNP A1 siRNA oligonucleotides, hnRNP C siRNA oligonucleotides, or a scrambled control (Sangon Biotech, China) were transfected into cells using RNAiMAX (Invitrogen, USA). After 48hr, cells were harvested. RNA isolation and RT-PCR analysis of *FBN1* genomic fragment were performed. Knockdown of hnRNP A1 and hnRNP C was validated by quantitative reverse transcription-polymerase chain reaction (qRT-PCR) tests.

**Bioinformatic analysis**

Both 1000 Genomes Project database (http://www.1000genomes.org) and Exome Variant Server database (http://evs.gs.washington.edu/EVS/) were used to retrieve the novelty of the point mutation. Additionally, Various bioinformatics websites such as ESEfinder 3.0,^43^ EX-SKIP, HOT-SKIP, SpliceAid2 and were applied to analyze the underlying mechanisms of pathological exon splicing event.
