# Supplementary material for: The abnormal splicing regulation network caused by synonymous mutations in FBN1 exon 39 leads to Marfan syndrome
Source: Genes Dis. 2024 Jul 3;12(3):101371. doi: 10.1016/j.gendis.2024.101371 (PMC11772951; doi:10.1016/j.gendis.2024.101371)
Supplement: Multimedia component 2 [file mmc2.docx]

**Supplementary figure captions**

**Figure S1.** The HOT-SKIP tool was used to predict hot spot mutations causing exon39 skipping. Dark red indicates the most likely mutation to contribute to exon skipping. The hottest pots are indicated with arrows (E+31A>T, E+32A>G and E+26A>G). The patient mutation (c.4773A>G) is one of the three predicted popular mutations, corresponding to the position of the red arrow (E+26A>G), and the other two predicted results are the positions of the blue arrow.

**Figure S2.** Three bioinformatics tools were performed to analyzed the binding spliced proteins in WT and MUT sequences. (a) MUT sequence produced an ESE-binding splicing protein SRSF1 using ESEfinder 3.0, comparing to the WT sequence. (b) The ratio of ESS/ESE changed from 0.87 to 1.56 after the mutation occurred using EX-SKIP. (c) MUT sequence bound more inhibitory splicing proteins using SpliceAid2. **Abbreviation:** WT, wild-type. MUT, mutant-type. ESE/ISE, exon/intron splicing enhancer.

**Figure S3.** Effects of hnRNP A1 and hnRNP C knockdown on splicing events. (a) and (b) qRT-PCR result shows hnRNP A1 and hnRNP C has been knocked down to a certain extent. (c) WB results indicated hnRNP C were knocked down.

**Figure S4.** The specific AONs are used to block the binding of splicing proteins. (a) Prediction of the most likely site where an AON might bind using SpliceAid2 tool. (b) The electrophoresis results showed a normal band (371bp), suggesting the mis-splicing had not been corrected by the AON.
